# The chinchilla animal model for hearing science and noise-induced hearing loss

**DOI:** 10.1121/1.5132950

**Published:** 2019-11-27

**Authors:** Monica Trevino, Edward Lobarinas, Amanda C. Maulden, Michael G. Heinz

**Affiliations:** School of Behavioral and Brain Sciences, Callier Center, The University of Texas at Dallas, 1966 Inwood Road, Dallas, Texas 75235, USA; Department of Speech, Language, and Hearing Sciences, Purdue University, 715 Clinic Drive, West Lafayette, Indiana 47907, USA; Weldon School of Biomedical Engineering, Purdue University, 715 Clinic Drive, West Lafayette, Indiana 47907, USA

## Abstract

The chinchilla animal model for noise-induced hearing loss has an extensive history spanning more than 50 years. Many behavioral, anatomical, and physiological characteristics of the chinchilla make it a valuable animal model for hearing science. These include similarities with human hearing frequency and intensity sensitivity, the ability to be trained behaviorally with acoustic stimuli relevant to human hearing, a docile nature that allows many physiological measures to be made in an awake state, physiological robustness that allows for data to be collected from all levels of the auditory system, and the ability to model various types of conductive and sensorineural hearing losses that mimic pathologies observed in humans. Given these attributes, chinchillas have been used repeatedly to study anatomical, physiological, and behavioral effects of continuous and impulse noise exposures that produce either temporary or permanent threshold shifts. Based on the mechanistic insights from noise-exposure studies, chinchillas have also been used in pre-clinical drug studies for the prevention and rescue of noise-induced hearing loss. This review paper highlights the role of the chinchilla model in hearing science, its important contributions, and its advantages and limitations.

## I. INTRODUCTION

The chinchilla, or more specifically the long-tailed chinchilla (*Chinchilla lanigera*), has been used as an animal model for studying auditory function and the effects of hearing loss for many decades, with references dating back to the early 1960s. Chinchillas are indigenous to the Andes Mountains of South America, where they live in herds. The name chinchilla means “little chinchas,” which is a reference to the Chincha people who lived in the same mountain range. Chinchillas were first domesticated when they were brought to the United States in the early 1920s by Mathias F. Chapman, a US-American mining engineer working in Chile. At that time, chinchillas were protected by the Peruvian, Bolivian, Chilean, and Argentine governments, so Chapman was granted special permission to bring back 11 chinchillas (three females) to the United States to begin a breeding population. Today, there are hundreds of chinchilla ranches in the US, some of which supply chinchillas for auditory research whereas others raise chinchillas for the furrier industry.

It is important to note that chinchillas are a protected species in the United States via the Animal Welfare Act (AWA) enacted in 1966; provisions to this act are enforced by the Department of Agriculture (USDA). Thus, research using chinchillas is strictly regulated and must be scientifically justifiable over lower species. A number of anatomical, physiological, and behavioral advantages do exist that justify the use of chinchillas for auditory research and have made it an extremely valuable animal model for hearing science. Primary among these advantages are the similarity of their hearing frequency range and sensitivity to humans and their genetic heterogeneity compared to other rodent models. Additionally, the chinchilla's docile nature and long lifespan (∼10 years in the wild; up to 15–20 years domesticated) make it suitable for short- or long-term studies. The ability to measure and relate a breadth of anatomical, behavioral, and physiological effects of various noise exposures within the same species has led to widespread use of the chinchilla as a model for noise-induced hearing loss (NIHL). The chinchilla's history as an animal model of acoustic injury has also led to a number of studies exploring pharmacological rescue and prevention using antioxidants and other biologically active compounds and drugs aimed at preventing or limiting the effects of noise trauma.

The overall aims of this paper are to (1) highlight the historical relevance of the chinchilla model in hearing-science research, including its strengths and limitations, (2) review the breadth of key findings related to noise exposure that demonstrate the usefulness of the chinchilla animal model, and (3) highlight some of the subsequent chinchilla studies evaluating the ability of pharmacological agents to protect against NIHL.

## II. CHINCHILLAS AS A GENERAL MODEL FOR HEARING SCIENCE

### A. Similarities with human hearing

**FIG. 1.**
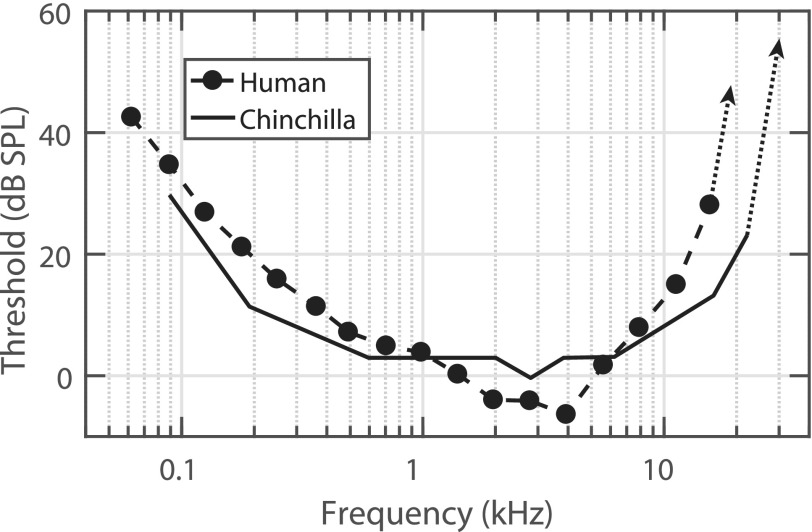
The hearing frequency range and sensitivity of chinchillas overlap significantly with human hearing, which is one of the main advantages of chinchillas as an animal model of hearing. Behavioral audiograms are compared between chinchillas and humans. Data replotted with permission from Miller (1970). Copyright 1970, Acoustic Society of America.

An important advantage that chinchillas have over many other rodent models is that their hearing sensitivity and frequency range significantly overlaps with humans (Fig. 1; Miller, 1970), with their average hearing range extending from ∼50 Hz to 33 kHz (Heffner and Heffner, 1991). The frequency-range similarity between chinchillas and humans is unlike the higher-frequency hearing of mice and rats. Thus, chinchillas are a particularly good rodent model for studies that use perceptually relevant stimuli for humans (e.g., speech).

Anatomical similarities with humans include a similar number of cochlear turns (humans: two and three quarters; chinchillas: three), a similar variation in cochlear length (range of the mean: humans: 33%; chinchillas: 26%), and a wide tympanic membrane (Bohne and Carr, 1979; Martin, 2012; Thornton *et al.*, 2013). One anatomical difference between humans and chinchillas is cochlear length, with humans at ∼31.5 mm and chinchillas at ∼18 mm (Hardy, 1938; Bohne and Carr, 1979).

The chinchilla's genetic diversity, in contrast to some mice strains used to study hearing, may also provide an advantage as a model of human hearing and disease (Salvi and Boettcher, 2008). This relatively broader genetic diversity stems from the fact that chinchillas are not inbred; instead, chinchillas are typically obtained from local chinchilla ranches. Thus, chinchillas may better mirror the diversity of biological responsiveness that would be encountered in human clinical trials. Conversely, if the research is aimed at specific genetic attributes or manipulations, then the chinchilla may be a less desirable choice to achieve the study's aims in comparison to mice. More recently, however, the chinchilla genome was sequenced in 2012, with the subsequent development of a database to compare the chinchilla and human genome (Shimoyama *et al.*, 2016). This recent advancement in mapping the genome could pave the way for more advanced chinchilla studies on specific genetic factors influencing NIHL.

### B. Behavioral, anatomical, and physiological advantages

The ability to make a wide range of measurements within the same species is a primary advantage of the chinchilla model. The chinchilla's temperament (e.g., docile nature, easy trainability) permits behavioral performance to be assessed using operant conditioning for simple and complex stimuli, as well as for physiological data collection from awake animals (e.g., middle-ear muscle reflexes). Likewise, anatomical and physiological characteristics in chinchillas have allowed for robust data collection along the entire auditory system using both invasive and non-invasive techniques. Thus, the widespread use of chinchillas in NIHL studies (Sec. III) has been largely derived from the ability to collect behavioral, anatomical, and physiological data from a single species. This ability has allowed researchers to garner a great depth of insight into underlying mechanisms of NIHL, as well as potential targets for pharmacological intervention (Sec. IV). In this section (Sec. II B), we review a number of studies and their key findings that highlight the important contributions of the chinchilla model to auditory research.

#### 1. Behavioral studies of hearing sensitivity and sound discrimination

Chinchillas are relatively easy to train and have been used extensively in behavioral studies of sound detection and discrimination. These studies include experiments on pitch/intensity detection and discrimination, signal detection in noise, temporal changes in acoustic cues, and sound localization. Their long lifespan relative to other rodents, combined with their intelligence, make long-term behavioral experiments feasible (e.g., pre- and post-exposure and/or treatment). Chinchillas can be used to measure both operant as well as reflexive responses to acoustic stimuli. Operant conditioning is a preferred method because once trained, the animals are reliable attending observers under stimulus control. This attention permits implementing the types of complex tasks that are necessary to test both threshold and suprathreshold hearing assessments pre- and post-intervention. Operant conditioning studies with chinchillas have used negative-reinforcement (e.g., shock avoidance) as well as positive-reinforcement approaches (e.g., behaviors reinforced by food). However, there are no peer-reviewed studies using reflexive measures in the chinchilla in response to acoustic stimuli (e.g., the startle response). It is possible that the paucity of startle studies in chinchillas is due to long release times from the startled state, or the presence of only subtle indicators of a startle response similar to those observed in the guinea-pig post-auricular response (Wilson *et al.*, 2019).

Early studies in the chinchilla were focused on hearing sensitivity and auditory perceptual changes in intensity, frequency, and time. Many of these studies used operant conditioning techniques that were widely available at the time to study sensory perception. These techniques predated the now routinely used electrophysiological measures used to assess hearing sensitivity in animals. One of the earliest such studies at the Central Institute for the Deaf in St. Louis used a shock avoidance technique to assess chinchilla free-field auditory thresholds (Miller, 1970). The findings showed remarkable similarities between chinchilla and human thresholds across a broad range of frequencies ranging from ∼0.1–10 kHz (Fig. 1). The successful use of operant techniques and the observation of human-like hearing sensitivity led to a number of subsequent important studies. For example, variations of the shock-avoidance technique were used to demonstrate the effects of prolonged noise exposure on temporary (TTS) and permanent (PTS) threshold shifts (Miller, 1970; Carder and Miller, 1972; Eldredge *et al.*, 1981), as well as the correlation between loss of sensory cells in the inner ear and elevated thresholds (Hunter-Duvar and Bredberg, 1974). Shortly thereafter, a positive-reinforcement procedure adapted from behavioral methods used in non-human primates (Stebbins *et al.*, 1973) was introduced to study the effects of noise exposure on auditory thresholds (Clark *et al.*, 1974). Chinchillas were trained to press a telegraph key when sound was presented in order to activate a feeder that delivered a 45-mg food pellet. The feeder type and pellet size were readily available and broadly used in rat behavioral research. The results from this positive reinforcement study showed thresholds similar to those reported by Miller (1970) at low frequencies, and slightly poorer thresholds at high frequencies. Although these methods yielded similar results, the use of these behavioral techniques often requires complex and time-consuming schedules for both learning and for performance assessment (Henderson *et al.*, 1983; Davis and Ferraro, 1984).

Behavioral techniques using shock avoidance can reduce overall training time, but these are not without disadvantages. For instance, animals that are shocked likely experience significantly more stress than those that are positively reinforced. Despite some limitations, behavioral techniques show that the chinchilla can be reliably trained to respond to a variety of acoustic stimuli relevant to studies of NIHL.

A main advantage of the aforementioned behavioral techniques is that these can be adapted to gather complex auditory perception information. Animals can be taught to detect both the presence of acoustic stimuli as well as *changes* in the acoustic stimulus. In a series of studies, the shock avoidance technique was used to assess temporal acuity in normal and hearing-impaired chinchillas (details reviewed in this special issue by Radziwon *et al.*, 2019). These studies were motivated by human studies showing poorer performance on temporal tasks in hearing-impaired listeners relative to their normal-hearing peers. Temporal acuity was evaluated in chinchillas using a gap detection task (Giraudi-Perry *et al.*, 1982), where performance was observed to degrade as a function of increasing threshold shift. These results were consistent with the prevailing models of temporal resolution at the time. It was also shown that, by varying the frequency content of the noise exposure, gap detection thresholds primarily depended on high-frequency information. Importantly, the pattern of deficits was similar to that observed in humans with high-frequency hearing loss.

An alternative to the gap-detection approach for assessing temporal resolution is to determine an amplitude-modulation detection threshold. This can be achieved by presenting a broadband stimulus and applying amplitude modulation at various frequencies as well as modifying modulation depth. In experiments on chinchillas, a shock-avoidance technique was used to assess amplitude-modulation detection thresholds (Henderson *et al.*, 1984). Similarly to the results found in the gap-detection studies, poorer temporal resolution was also found to depend primarily on high-frequency hearing.

Several studies have evaluated the relative discrimination abilities between chinchillas and humans with respect to changes in either frequency or intensity. Similar to temporal acuity studies, the patterns of discrimination performance were similar between chinchillas and humans. However, in contrast to the only slightly poorer temporal acuity seen in chinchillas, discrimination performance was substantially worse for chinchillas compared to humans. In an operant go-no-go shock-avoidance frequency-discrimination threshold task, chinchillas showed that even after considerable training, only large frequency changes could be detected, particularly for low frequencies (Nelson and Kiester, 1978). When the task was simplified, chinchilla performance improved, but differential sensitivity remained approximately ten times poorer than observed in humans. Similarly, with a food-reward operant frequency-discrimination task using simple and complex tones, chinchillas showed similar patterns to humans (lower thresholds for complex tones than for single tones), but overall worse frequency-discrimination ability (Shofner, 2000). Intensity-discrimination studies found larger difference limens in chinchillas relative to humans. In studies using shock avoidance or positive reinforcement (Saunders *et al.*, 1987; Graf *et al.*, 1992), functions between humans and chinchillas were similar in shape but chinchillas required considerably larger intensity differences. A positive-reinforcement operant study of increment detection in band-limited noises also showed that performance in chinchillas and humans has a similar dependence on total bandwidth, but on average, chinchillas required larger increments for successful detection (although some chinchillas showed human-like thresholds, Shofner *et al.*, 1993). These differences suggest that, although hearing sensitivity and temporal resolution are similar between humans and chinchillas, overall, chinchillas appear to be less sensitive in differential discrimination, particularly in the intensity and frequency domain.

#### 2. Auditory brainstem responses (ABRs)

**FIG. 2.**
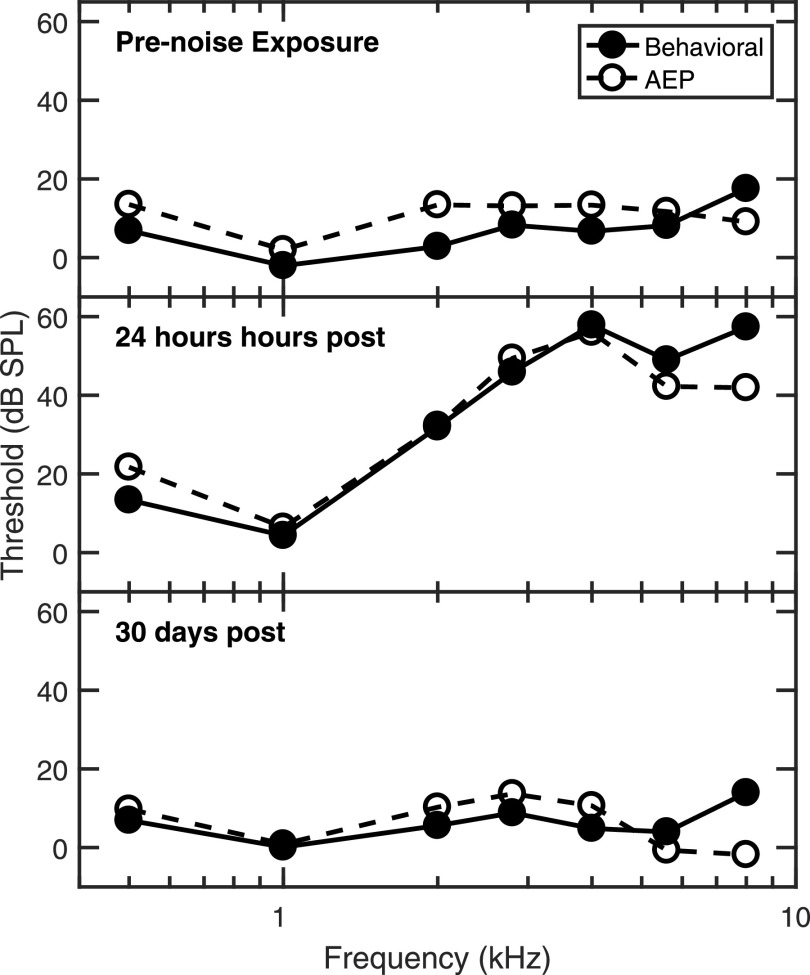
Hearing sensitivity thresholds derived from easier-to-measure auditory evoked potentials (AEPs) match well with behavioral thresholds for both normal-hearing and noise-exposed chinchillas. Behavioral and AEP thresholds measured from the same three chinchillas before, one day after, and 30 days after noise exposure showed no statistically significant differences. The similarity of AEP and behavioral thresholds demonstrates that AEPs provide a time-efficient alternative to behavioral threshold measures that allows for much more detailed studies of the effects of noise exposure on hearing. Data from individual animals in Henderson *et al.* (1983) were averaged and replotted with permission from the International Journal of Audiology.

The studies presented in Sec. II B 1 demonstrate the ability to assess a wide range of threshold and suprathreshold auditory functions in chinchillas with relatively minimal (shock avoidance) to moderate training (positive reinforcement). However, behavioral studies of any type are more difficult and time consuming than objective electrophysiological studies. As auditory evoked potential (AEP) measurement systems were made more readily available and more sophisticated in the early 1970s, the behavioral techniques described were less often used in experiments where the outcomes were simple measures of audibility (e.g., before and after noise exposure). ABRs have been shown to agree closely with behavioral techniques for both normal-hearing and hearing-impaired animals (e.g., Fig. 2; Henderson *et al.*, 1973; Henderson *et al.*, 1983). Furthermore, ABR threshold elevations are well correlated with auditory-nerve (AN) fiber threshold elevations in noise-exposed chinchillas and decreases in ABR wave-1 latency are correlated with decreases in AN-fiber frequency selectivity (Henry *et al.*, 2011). The advantage of using reliable non-invasive objective measures that require no training cannot be understated.

#### 3. Otoacoustic emissions (OAEs)

ABRs and other AEPs have been used extensively to measure thresholds as a function of frequency and consequently to evaluate frequency-specific changes in thresholds after noise exposure. A common secondary assay of cochlear damage is distortion product otoacoustic emission (DPOAE) testing, a robust measure in chinchillas related to the non-linear gain provided by outer hair cells (OHCs). In chinchillas, a linear relation between DPOAE amplitude and percentage of OHC loss was established even when OHC lesions were relatively broad (Hofstetter *et al.*, 1997). DPOAEs were also successful in identifying broad regions of permanent OHC loss (>10%) as well as focal lesions extending more than 0.6 mm in noise-exposed chinchillas (Harding *et al.*, 2002). Functionally, DPOAE amplitude shifts in noise-exposed chinchillas were strongly correlated with ABR and AN-fiber threshold shifts, and weakly correlated with AN-fiber frequency selectivity (Henry *et al.*, 2013). Thus, interpretations of DPOAE thresholds and amplitudes provide useful, but still limited, information as DPOAEs are indirect measures of OHC integrity and function (Shaffer *et al.*, 2003). Alternatively, studies have used other types of OAEs, such as stimulus-frequency OAEs (SFOAEs), to suggest (with some controversy) that human cochlear tuning is exceptional sharp with respect to laboratory animals (e.g., Shera *et al.*, 2002; Ruggero and Temchin, 2005). Auditory-nerve-fiber and SFOAE data from chinchillas have been used to validate the use of SFOAEs to estimate cochlear tuning, and thus to support the notion of sharper tuning in humans (Shera *et al.*, 2010). Taken together, OAE studies in the chinchilla can be extremely valuable in determining the effects of NIHL on OHC loss, cochlear non-linearity, and frequency selectivity.

#### 4. Awake physiological measures of auditory function

The chinchilla's docile nature and durability to chronic surgeries provide further significant advantages that allow for a wide range of physiological measurements to be made from restrained awake animals (Snyder and Salvi, 1994). For example, awake AEPs have been recorded from chronically implanted electrodes in the inferior colliculus (IC) for a number of purposes. To evaluate the role of the pinna and ear canal in determining frequency-dependent sensitivity to noise exposure, head-related transfer functions were measured and compared to AEP-based audiograms in chinchillas measured with both free-field acoustic stimulation and insert earphones (Murphy and Davis, 1998). These data demonstrated that pinna and ear-canal filtering provide up to 10-dB of gain around 4 kHz, which underlies the hypersensitivity of chinchillas (and other species) to mid-frequency noise exposures (Rosowski, 1991). Chronic IC AEPs have also been measured before and after permanent NIHL to evaluate physiological forward-masking patterns in comparison to psychophysical patterns in humans (e.g., Arehole *et al.*, 1989).

Electrophysiological measures from the round window during long-duration noise exposure have also been made to evaluate the protective nature of the middle-ear muscle reflex (MEMR; Gerhardt *et al.*, 1980). In particular, the ability to measure tympanometry and acoustic reflexes in awake animals is critical because middle-ear impedance is affected by anesthesia in chinchillas (Woodford *et al.*, 1975), and the MEMR is eliminated by anesthesia (Woodford *et al.*, 1976). The MEMR has recently been proposed as a reliable assay of cochlear synaptopathy, a loss of inner hair cell (IHC) afferent synapses, that can follow moderate level noise exposures (Valero *et al.*, 2018). The ability to perform these types of measurements on awake chinchillas continues to represent an important advantage of this animal model.

#### 5. Ease of access to the middle ear and cochlea

Chinchillas have large heads with relatively easy access to the middle ear and cochlea via an enlarged auditory bulla. This advantage has led to a breadth of detailed published data on normal middle-ear and cochlear function, device development, and the effects of conductive and peripheral hearing losses (Martin, 2012). The readily accessible middle-ear space and cochlea allow for complex experimental manipulations to quantify sound transmission through the middle ear and into the cochlea (Rosowski *et al.*, 2006; Slama *et al.*, 2010; Ravicz and Rosowski, 2013; Robles *et al.*, 2015; Wang and Gan, 2016; Peacock *et al.*, 2018). Furthermore, easy cochlear accessibility allowed for thorough and rigorous characterizations of cochlear-nonlinearity properties (i.e., level-dependent gain and bandwidth effects in basilar-membrane responses) to be collected following delicate microsurgical procedures that can easily damage the normal function of the cochlea (Robles *et al.*, 1997; Ruggero *et al.*, 1997; Rhode and Recio, 2000). These detailed data on cochlear nonlinear response properties and their relation to healthy cochlear function have provided significant insight into perceptual consequences of cochlear hearing loss (Oxenham and Bacon, 2003). The anatomical advantages of the chinchilla have also led to a number of important studies on device development (Del Socorro Hernandez-Montes *et al.*, 2009; Lupo *et al.*, 2011; Lupo *et al.*, 2012; Chang *et al.*, 2013). A final advantage of the chinchilla ear is the ease of access and physical thinness of the round window, which permits near-field electrophysiological measures of cochlear function (Durrant *et al.*, 1998; Burkard *et al.*, 1999) and more effective drug penetration into the cochlea (Martin, 2012).

#### 6. Robust systemic physiology allows single-unit neural coding studies

The robustness of chinchillas to surgical procedures and barbiturate anesthesia allows for lengthy (∼24–36 h) neurophysiological single-neuron experiments (e.g., Kale and Heinz, 2010). Due to their robustness, there is a wealth of detailed data characterizing neural responses to both simple and perceptually relevant complex sounds across the entire peripheral and central auditory system in the chinchilla. This is particularly true for the auditory nerve, for which fundamental questions in hearing science regarding frequency selectivity and temporal responses have been addressed.

An enormous dataset characterizing anesthetized chinchilla AN-fiber tuning properties (>4,000 AN fibers; Temchin *et al.*, 2008a,b) addressed the physiological mechanisms underlying frequency selectivity by comparing neural data with the thorough mechanical basilar-membrane data also available from chinchillas (Ruggero *et al.*, 1997). The anatomical advantages of the chinchilla have allowed tuning data to be collected simultaneously from the basilar membrane and AN fibers (Narayan *et al.*, 1998). These invaluable data showed a very close match between mechanical and neural tuning in the same cochleae, suggesting that the only significant contributor to mammalian cochlear tuning is basilar-membrane mechanics (i.e., refuting the need for a “second” filter, a long-standing debate in the field).

Extensive AN-fiber studies in the chinchilla have also examined fundamental questions related to temporal processing. These include the dependence of threshold on stimulus duration, results that have been shown to be similar to those obtained with psychophysical measures (Eddins *et al.*, 1998). Other experiments have elucidated changes in temporal dynamics of AN-fiber responses following noise exposure (Scheidt *et al.*, 2010). These experiments have led to a better understanding of neural coding of modulation, such as the role that cochlear nonlinearity may play in preserving AN-fiber modulation coding in noise (Frisina *et al.*, 1996). The enhancement of AN-fiber envelope coding following noise exposure (despite no change in the fundamental ability of AN fibers to follow temporal fine structure) was shown to be consistent with perceptual studies, revealing the complexities of the perceptual correlates incurred by noise exposure (Kale and Heinz, 2010). Chinchilla studies have used complex speech-like stimuli to study AN-fiber coding, providing important insights for understanding the effects of hearing loss on speech perception in hearing-aid and cochlear-implant users (Sinex *et al.*, 2003; Loebach and Wickesberg, 2006; Heinz and Swaminathan, 2009). Changes in coding of speech-like stimuli that occur as the AN-fiber representation is processed in the cochlear nucleus have also been studied in the chinchilla (Rhode, 1998), as well as modulation coding in the IC (Langner *et al.*, 2002).

As sound travels further up the central auditory system, inputs from both ears are integrated and used to perform higher-order tasks such as sound localization along the horizontal and vertical planes. The role of the head and pinna in creating the binaural acoustic cues used for spatial hearing (i.e., interaural level and time differences, ILDs and ITDs, and monaural spectral shape cues) has been studied in chinchillas in terms of the head-related transfer functions (Jones *et al.*, 2011; Koka *et al.*, 2011). Binaural interactions below the level of the superior olive have been demonstrated in chinchillas (Mast, 1970). Specific processing details within the binaural circuits of the lateral superior olive have been explored through extracellular and intracellular studies (Moore and Caspary, 1983; Finlayson and Caspary, 1989). Finally, studies of binaural coding in chinchillas have examined the important interaural cues underlying sound localization, including ITDs in the medial superior olive (Langford, 1984; Bremen and Joris, 2013) and ILDs in the lateral superior olive and IC (Finlayson and Caspary, 1991; Jones *et al.*, 2013; Jones *et al.*, 2015; Brown and Tollin, 2016).

Supplementing these higher-order findings, data from auditory-cortex neurons in chinchillas were used to explore which aspects of sensitivity to FM sweeps, a fundamental building block of speech, are experience-dependent in development (Brown and Harrison, 2011). The ability to record large datasets of perceptually relevant complex stimuli from various locations along the auditory system in the same species is essential, as the effects of NIHL likely reflect both peripheral and central changes.

Furthermore, important and significant effects of NIHL have been shown to be dependent not only on the recording site, but also on the acoustic stimulus used. For instance, unlike broadband-noise stimuli, narrowband stimuli can fail to capture the distorted tonotopic organization often found following noise exposure (Henry and Heinz, 2013; Henry *et al.*, 2016). The ability to perform experiments along the entire auditory pathway in the same species with relevant acoustic stimuli presents a number of opportunities to leverage the strengths of the chinchilla model to address fundamental and mechanistic consequences of hearing loss for both monaural and binaural processing.

### C. Disadvantages of the chinchilla model for hearing science

The use of the chinchilla for research purposes is not without its own unique challenges. Most notably, the chinchillas used for research in the United States are acquired from select ranches whose main purpose is to breed them for their fur. Often, animals sold to researchers are considered undesirable to furriers due to fur quality. Poor fur quality can indicate potential underlying health concerns; however, major health concerns are rare. For example, fur chewing is often an indicator of elevated animal anxiety. In a lab setting, this can generally be remedied by adding additional enrichment for the animal. For longer-term studies (e.g., behavior and/or noise-exposure studies), underlying health problems can create unexpected complications and potential attrition that may occur more frequently than with other common research rodents. Furthermore, due to their less frequent use in research, veterinarians and their staff are generally less experienced with chinchilla medical care than other rodent species. This limitation can result in difficulty with early detection of illness and prescribing appropriate treatment. Under many circumstances, chinchillas require care by an exotic animal veterinarian. It should be noted, however, that despite these potential limitations, many long-term studies of behavior and noise exposure have been carried out successfully in chinchillas, as discussed throughout this review.

Another important consideration is that chinchillas must be housed in a manner that differs from most laboratory rodents. For example, because chinchillas have 60–90 hairs per follicle, they are at higher risk for overheating and must be kept at cool temperatures and low humidity levels. Ideally, chinchillas should be kept in housing conditions that resemble their native mountain habitat (65 °F, less than 60% humidity). Temperatures above 75 °F can become fatal. Compared to other rodents used in research, such as mice and rats, chinchillas also require more elaborate diets that are rich in fiber and protein in order to maintain healthy gastrointestinal systems. Chinchillas also routinely require dust baths to absorb oils and moisture buildup in their fur. These baths mimic dust available in their natural habitat that helps keep their fur clean and healthy.

It is also important to note that the chinchilla's 112-day gestation period is substantially longer than the 21-day gestation period for rats and mice. Furthermore, one gestational period only yields 1–2 kits. Weaning a chinchilla kit takes about six weeks, compared to rats and mice that only require three weeks. These limitations make it much more difficult to breed and raise chinchillas in research studies for which well-controlled acoustic environments are desired from birth. Chinchillas are also born with a fully developed auditory system, which may or may not be advantageous to the research of interest and could significantly limit developmental studies of the auditory system.

Anatomically, chinchillas have a thin bulla and skull. Though this allows easy access to the cochlea and other structures, it can also create a challenge for mounting implanted chronic electrodes and electrode arrays. Differences in outer and middle ear acoustics may contribute to differences in noise susceptibility between chinchillas and other species (Drescher and Eldredge, 1974). Chinchillas are generally much more susceptible to noise damage than many other species, including humans and monkeys (Luz and Lipscomb, 1973; Dobie and Humes, 2017; Valero *et al.*, 2017); however, the general mechanisms of noise-induced damage appear to be similar across mammals (Mills *et al.*, 1979). Liver metabolism in chinchillas also differs from other rodents, which may contribute to the differential hearing loss observed between rats and chinchillas with drug induced hearing loss or in studies where ototoxic drugs potentiate NIHL (Davis *et al.*, 2002). This difference in metabolism is also important to consider, as it relates to pharmacologic oto-protective treatments for NIHL.

Finally, when considering the use of chinchillas for research purposes, it is crucial to remember their USDA status as a protected species. Per the AWA, the use of chinchillas must be scientifically justifiable over lower species, which can make approval from Institutional Animal Care and Use Committees more difficult than for other rodent species.

### D. Models of hearing loss

In addition to studies of NIHL (described in Sec. III), the chinchilla has also been used to study other types of hearing loss that provide insight into the contribution of mechanical and metabolic insults to hearing deficits. Given the aforementioned physical attributes of the chinchilla, these studies can be leveraged to study specific injuries to middle-ear structures.

#### 1. The middle ear

The chinchilla is considered a gold standard animal model for various diseases of the middle ear, including otitis media, stapes fixation, and cholesteatoma (Lupo *et al.*, 2009; Martin, 2012; Guan *et al.*, 2014; Guan *et al.*, 2015; Wang and Gan, 2018). Chinchillas have also been used to evaluate the strength of various diagnostic tests of middle-ear function (e.g., multifrequency tympanometry has been shown to provide information that is not available in the 226-Hz tympanogram; Margolis *et al.*, 1995). In an experimental model of otitis media with effusion, the middle-ear space in chinchillas was systematically filled with fluid to evaluate the effects of conductive pathology (Thornton *et al.*, 2013). These types of studies provide opportunities to study underlying mechanisms and consequences of middle-ear disease commonly reported in humans. The increasing knowledge of middle-ear sound transmission in chinchillas with normal hearing and conductive losses (e.g., Rosowski *et al.*, 2006; Chang *et al.*, 2013; Wang and Gan, 2016) are also extremely valuable in interpreting blast-induced hearing losses. In these cases, rupture of the tympanic membrane has often been associated with less sensory hearing loss in chinchillas (Hamernik *et al.*, 1984; Hickman *et al.*, 2018) due to the conductive loss limiting sound energy to the inner ear, as discussed in more detail in Sec. III E.

Chinchillas have also been used as an animal model to study otopathology and bone conduction of sound. A series of studies on the effects of superior semicircular canal dehiscence on hearing sensitivity in chinchillas has provided useful experimental and theoretical insight (Rosowski *et al.*, 2004; Songer and Rosowski, 2005, 2007). Air-bone gaps at low frequencies were created when a dehiscence was made in the superior canal of the chinchilla (Songer and Rosowski, 2010). These data support the third-window hypothesis regarding how a dehiscence can produce a conductive hearing loss by shunting energy away from the cochlea and were qualitatively consistent with data from human patients. Several chinchilla studies have investigated the roles of the middle and inner ears to bone conduction (Chhan *et al.*, 2013; Chhan *et al.*, 2016). An osseointegrated bone-conducting prosthesis has been studied in chinchillas for translational application to sound localization in cases of single-sided deafness (Tringali *et al.*, 2015).

#### 2. The inner ear

Noise-induced hearing loss is typically associated with damage to both OHCs and IHCs (Liberman and Dodds, 1984). Because these two types of hair-cell damage sometimes produce confounding effects on neural responses, interpretation of the physiological correlates of perceptual effects following noise exposure can be difficult (e.g., Heinz *et al.*, 2005).

**FIG. 3.**
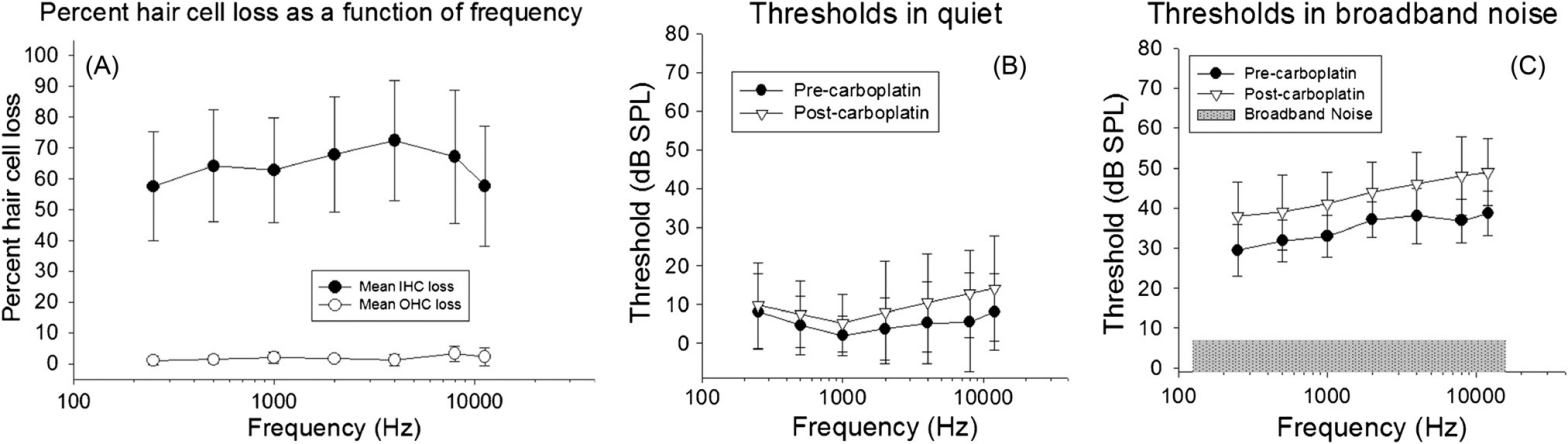
Chinchillas have been used to demonstrate that audiograms (detection of tones in quiet) can be insensitive to significant IHC loss that causes suprathreshold deficits (e.g., detection of tones in noise). (A) Carboplatin can cause selective IHC loss in chinchillas. (B) IHC loss did not affect behavioral pure-tone detection in quiet, but (C) did degrade tone-in-noise detection, demonstrating an additional model of “hidden hearing loss” to the more commonly discussed model with cochlear synaptopathy. Figures reprinted from Lobarinas *et al.* (2016), with permission from Springer Nature (JARO).

Chinchillas provide a unique model for understanding the effects of selective IHC dysfunction. Unlike other species where IHC and OHC dysfunction often occur together, in chinchillas carboplatin (a platinum-based chemotherapy drug) can be used to produce selective IHC damage uniformly along the cochlea [e.g., Fig. 3(A); Wake *et al.*, 1994; Trautwein *et al.*, 1996; Wang *et al.*, 1997]. The pattern of damage includes both IHC death as well as IHC dysfunction due to ultra-structural stereocilia damage (Wake *et al.*, 1994). Chinchilla AN-fiber responses following carboplatin typically have normal tuning with reduced spontaneous and driven discharge rates, consistent with IHC stereocilia damage (Wang *et al.*, 1997). DPOAEs are unaffected in chinchillas with severe (>80%) carboplatin-induced IHC loss when OHCs are intact (Trautwein *et al.*, 1996). Perceptually, the carboplatin chinchilla model was used to demonstrate that the behavioral audiogram was insensitive to less than 80% IHC loss [Fig. 3(B); Lobarinas *et al.*, 2013]. In contrast, suprathreshold sensitivity to tones in noise was diminished in this model [Fig. 3(C)], despite functioning OHCs and normal frequency selectivity (Lobarinas *et al.*, 2016). The unique carboplatin chinchilla model provides important insights into noise-induced perceptual effects by demonstrating that suprathreshold listening-in-noise difficulties can occur as a result of IHC dysfunction, even in the absence of permanent audiometric threshold shifts (Lobarinas *et al.*, 2013). Thus, IHC dysfunction and loss should be considered in interpreting noise-induced perceptual deficits in cases of both PTS and TTS.

The carboplatin model provides a complement to the selective OHC-loss models that antibiotics produce in many species, including chinchillas (Axe, 2017). For example, kanamycin selectively damages OHCs in chinchillas and produces elevated thresholds, broadened tuning, and loss of cochlear nonlinearity (e.g., Dallos and Harris, 1978). Each of these commonly measured effects is also observed following noise exposures that cause PTS. Thus, the selective OHC-loss model provides important insights into the underlying causes of the most commonly discussed effects of permanent NIHL (Axe, 2017).

Beyond the use of ototoxic drugs to create selective hair-cell models to dissect the effects of NIHL, chinchillas have also provided a useful animal model for testing specific drugs for ototoxicity. Alternative antibiotics have been tested for ototoxicity in chinchillas when the widespread use of non-ototoxic antibiotics has led to the development of antimicrobial resistance (Daniel *et al.*, 2007). Testing of broad-spectrum antibiotics thought to be useful as a topical agent for treating otorrhea has been performed in chinchillas (Brown *et al.*, 1989). Potential interactions between nonsteroidal anti-inflammatory drugs and noise exposure on chinchilla hearing sensitivity have also been studied (Woodford *et al.*, 1978). These examples demonstrate the usefulness of the well-established chinchilla model in studying ototoxicity and potential interactions with noise exposure.

#### 3. Metabolic models of hearing loss based on reduced endocochlear potential

Reduced endocochlear potential as a result of furosemide administration has been shown to be a model of metabolic presbycusis (Schmiedt *et al.*, 2002). Furosemide has been used in chinchillas to evaluate the effects of reduced endocochlear potential (i.e., a drained cochlear battery) on mechanical and neural responses (Ruggero and Rich, 1991; Henry *et al.*, 2019). Cochlear gain and frequency selectivity were greatly reduced following furosemide administration in chinchillas, consistent with reduced OHC function (Ruggero and Rich, 1991). The relative effects of metabolic and NIHL were compared in chinchilla AN-fiber responses to broadband noise, with an equal degree of NIHL creating a greater disruption in cochlear tonotopicity than a metabolic hearing loss (Henry *et al.*, 2019). The ability to directly compare two sensorineural hearing loss (SNHL) etiologies in the same species provides insight into unique aspects of NIHL that appear to be particularly significant for the neural coding of complex sounds, such as speech.

## III. CHINCHILLAS AS A MODEL FOR NOISE-INDUCED HEARING LOSS

Since the advent of the Industrial Revolution, humans have been exposed to louder and more varied noise over longer periods of their day. Today, two leading causes of hearing loss in humans are aging and noise exposure (Hoffman *et al.*, 2017). Approximately 22 million workers in the United States are exposed to hazardous noise that puts them at risk for occupational hearing loss, the most common work-related illness in the US (Masterson *et al.*, 2015). Without appropriate hearing protection, employees in industries such as mining, manufacturing, and construction are particularly at risk due to the intensity and duration of the noise they are exposed to within a typical work day. Though there are protections put in place by the Occupational Safety and Health Administration (OSHA), their recommendations are not always enforced or followed by workers. Some industries are required to monitor their employee's hearing through sequential hearing tests in order to make adjustments to the environment where possible and promptly provide rehabilitation when PTS is detected (OSHA, 1983).

Studies that examine the effects of noise exposure resulting in TTS as well as PTS have deep roots in the chinchilla model. In fact, chinchilla models of NIHL have been used by the National Institute for Occupational Safety and Health (NIOSH) to determine occupational-exposure guidelines for noise (NIOSH, 1972; Dunn *et al.*, 1991). While the majority of chinchilla studies are found in the peer-reviewed literature, a number of important studies are also found in government technical reports. In particular, a substantial amount of chinchilla hearing loss research has been conducted by the U.S. Army, in particular the US Army Aeromedical Research Laboratory (USAARL), and through contracts funded by the USAARL. Much of this work has been published in peer-reviewed literature; however, additional results are available as technical reports on the USAARL,^1^ and Defense Technical Information Center^2^ websites. This section (Sec. III) will highlight a number of the important contributions that chinchilla studies have made to further our understanding of the effects and mechanisms of NIHL.

### A. Anatomical effects of noise exposure

Many anatomical studies in the chinchilla have evaluated the degeneration of sensory hair cells in the inner ear as a function of noise trauma (e.g., Eldredge *et al.*, 1973; Bohne *et al.*, 1987; Boettcher *et al.*, 1992; Hamernik *et al.*, 1993). The effects of noise trauma typically involve mechanical and/or metabolic damage to OHCs from exposure to high levels of noise. For example, in a study evaluating the effects of acute to chronic (from 2 to 432 days) low-frequency [octave-band noise centered at 0.5 kHz, 47 or 95 dB sound pressure level (SPL)] or high-frequency (octave-band noise centered at 4 kHz, 47 or 95 dB SPL) noise exposures, chinchillas were found to develop significant losses of OHCs commensurate with the frequency content of the noise (Bohne and Harding, 2000). When exposed to high intensity, high frequency noise, chinchillas developed focal lesions in the cochlear region associated with 4–8 kHz. Prolonged exposure to the high-frequency noise destroyed entire segments of OHCs as well as myelinated afferent AN fibers in more apical regions of the cochlea. Prolonged exposure to the high-intensity, low-frequency noise created a lesion along the apex of the cochlea as expected. With prolonged exposure, however, the lesion extended from the apex to the base of the cochlea such that the damage profile towards the basal end was essentially indistinguishable from that observed following exposure to the high-frequency noise. The effects observed in these noise-exposed chinchillas mirror the profiles of damage seen in humans exposed to high levels of either low- or high-frequency noise. A more recent study looked at the post-noise time course of cochlear degeneration in 199 noise-exposed chinchilla ears and 22 controls (Bohne *et al.*, 2017). As expected, OHCs began to die during and soon after the exposure. However, longer-term data suggest that although OHC death slowed by one month, cochlear OHC degeneration continued for months later. In addition, there was a delayed, but significant degeneration of IHCs one-to-three weeks post-exposure as well as degeneration of supporting cells. These anatomical studies demonstrate the type of valuable insight that can be garnered from the chinchilla model of NIHL.

Other studies using less-intense noise exposures on chinchillas highlight an additional form of micro-structural cochlear damage that can occur following noise exposure. Slepecky *et al.* (1981) used chinchilla cochleas from a previous impulse-noise experiment where significant OHC loss did not translate to threshold shifts (Hamernik *et al.*, 1980). A scanning electron microscope allowed the researchers to observe the state of the OHCs and IHCs and found that, in addition to missing OHCs and IHCs, the remaining OHCs and IHCs in the damaged cochleas had deformed stereocilia. The permeability of the stereocilia membrane, surface charge, and actin conformation were all affected. More recent findings have confirmed that roughly equal degrees of OHC and IHC stereocilia damage can occur following noise-induced PTS (Boettcher *et al.*, 1992; Salvi and Boettcher, 2008), similar to results from more detailed structural-functional studies in cats (Liberman and Dodds, 1984), suggesting this effect is a more general mammalian consequence of noise exposure. This observation indicates that even though a hair cell may be considered “present” after noise exposure, damaged stereocilia can disrupt its function and can ultimately lead to PTS.

Recent immunohistochemical studies in mice have demonstrated that moderate-noise-exposures that produce a TTS can produce permanent cochlear synaptopathy, which is a degeneration of afferent synapses that may contribute to a latent form of hearing loss characterized by difficulties listening in noise despite an otherwise normal audiogram (Kujawa and Liberman, 2009). The same form of cochlear synaptopathy has been demonstrated to occur across a broad cochlear range in chinchillas following lower-frequency continuous-noise exposures that only produce TTS (Hickox *et al.*, 2017). Blast-induced cochlear synaptopathy has also been demonstrated in chinchillas, where synaptopathy was more focal in the middle and basal parts of the cochlea (Hickman *et al.*, 2018). The behavioral advantages of the chinchilla model described previously provide great potential to evaluate perceptual consequences of cochlear synaptopathy by allowing direct correlations between behavioral and anatomical consequences of various forms of NIHL.

### B. Non-invasive physiological correlates of noise-induced hearing loss

Noise-exposure studies in chinchillas have demonstrated that high-level exposures that cause hair-cell loss also decrease behaviorally derived estimates of hearing sensitivity (e.g., Clark *et al.*, 1974). The functional effects of hearing loss are the result of trauma to sensory cells in the inner ear, disruption and degradation of the neural signal in the auditory pathway, and behavioral threshold elevation (e.g., Henderson *et al.*, 1983). The relationship among hair-cell loss, threshold changes measured by auditory evoked potentials, and DPOAE changes has been well documented in chinchillas. In one study using acute noise exposures (center frequency: CF = 4 kHz, 108 dB SPL, 1–1.75 h or 80–86 dB SPL for 24 h), scattered and diffuse OHC loss (up to 10%) had no effect on DPOAE level shift, suggesting that loss of OHCs can precede measurable hearing deficits (Harding *et al.*, 2002). Another interesting finding was that threshold shifts best correlated with IHC and afferent fiber loss whereas small focal losses of OHC (100% over 0.4 mm, i.e., ∼2% of the ∼18 mm cochlea) had no effect on thresholds or DPOAEs. In a second study using chronic and repeated (1/s) impact noise (0.5–8 kHz, 400 Hz bandwidth, 109–127 dB SPL, 24 h/day for 5 days), 95 chinchillas were exposed to 23 different noise conditions (Hamernik and Qiu, 2000). The overall effects showed that when PTS was less than 25 dB, correlations among DPOAEs, ABR thresholds, and hair-cell loss were unremarkable. However, for larger threshold shifts, the relationship among the three variables was considerably better. The results of both these studies indicate that anatomical loss precedes measurable hearing impairment and that moderate to large hearing losses correlate well with both DPOAE measures and hair-cell loss. Importantly, these patterns appear to be similar to those found in humans exposed to various noise traumas that produce PTS.

In addition to elevated thresholds, hearing loss also produces other functional deficits. One of the most common is the loss of tuning, i.e., poorer frequency selectivity. Reduced frequency selectivity is thought to underlie the ability to hear in competing background noise (Moore, 1985). This masking of an acoustic target by a secondary sound stimulus can be studied in the time domain as well as the frequency domain. The audibility of a target stimulus can be studied by presenting a simultaneous competing stimulus or by presenting a masker stimulus right before or after a target and evaluating the effect of recovery from masking.

The effects of noise trauma on tuning characteristics (measured using simultaneous tone-on-tone masking) have been studied in chinchillas with evoked potentials assessed along the auditory pathway (Davis *et al.*, 1989; Ahroon *et al.*, 1993; Davis *et al.*, 1993). In two experiments, AEPs were measured from the IC before and after noise trauma from impulse, impact, or continuous noise that ranged in duration from acute, high-intensity (165 dB SPL) exposures to long-term (five days), low-intensity (80 dB SPL) exposures. In the first study, thresholds and tuning curves were measured in 150 chinchillas before and after noise exposure (Davis *et al.*, 1989). The main findings indicated that (1) thresholds increased as a function of noise-exposure duration and level, (2) small threshold shifts had no effect on tuning, and (3) as threshold shifts exceeded 10 dB, there was a progressive loss of tuning that was associated with OHC loss. The second experiment used a larger cohort of 363 chinchillas to evaluate the effects of noise trauma on thresholds and tuning (Ahroon *et al.*, 1993; Davis *et al.*, 1993). When PTS levels were low, tuning curves generally retained their shape. However, the low-frequency slope of the tuning curve provided evidence of noise-induced trauma even when animals had very little hearing loss. In both studies, high-intensity noise led to both significant PTS and degraded tuning, regardless of noise type. Furthermore, a detailed analysis of the tuning curve revealed that this metric can be used to detect the presence of hair-cell damage even when there is little evidence of hearing loss. Collectively, these studies demonstrate that the pattern of hearing loss and resulting deficits in chinchillas are similar to those observed in humans.

Forward-masking paradigms have shown that humans with hearing loss show poorer performance relative to individuals with no hearing loss (e.g., Smiarowski and Carhart, 1975). These experiments add a secondary level of complexity by involving a temporal component to masking. Forward masking can be studied using either behavioral methods or with advanced evoked potential measures. In chinchillas, forward-masking time constants ranged from 50 to 90 ms (Arehole *et al.*, 1989). However, following prolonged noise exposure (2-kHz tone, 105 dB SPL for five days), these time constants nearly doubled, particularly as PTS exceeded 20–25 dB. The magnitude of the change in time constant was consistent with threshold elevation, but was only weakly related to hair-cell loss. The pre- and post-exposure time constants were similar to those observed in humans with normal hearing and with hearing impaired individuals, respectively.

### C. Diagnostic potential of non-invasive physiological measures

The techniques shown thus far have demonstrated that the time course, threshold shifts, anatomical, and physiological changes in chinchillas exposed to noise can serve to better understand hearing loss in individuals. Furthermore, the chinchilla animal model can serve to develop more accurate diagnostic protocols to quantify and detect subclinical hearing loss, as well to predict underlying pathology. In a study evaluating the ABRs of chinchillas before and after noise exposure (115 dB SPL, narrow-band noise centered at 2 kHz, 50-Hz bandwidth, for 4 h), results showed that non-invasive methods could predict both the frequency selectivity and threshold responses of afferent AN fibers (Henry *et al.*, 2011). When compared to recordings from single AN fibers, ABR wave-1 threshold elevations were correlated with increases in AN-fiber threshold, whereas decreases in ABR wave-1 latency at equal sensation level (SL) were correlated with broadened tuning of AN fibers (i.e., loss of frequency selectivity). In a similar dataset, changes in DPOAE amplitudes were well-correlated with AN-fiber threshold shift near stimulus frequency, with the strongest correlation being for cochlear function near the *F*2 frequency (Henry *et al.*, 2013). Correlations with AN tuning were weaker than for threshold, but reached statistical significance. Recently, a non-invasive wideband MEMR assay of cochlear synaptopathy in awake chinchillas was shown to have large and consistent reductions in suprathreshold amplitudes following a noise exposure that only produced a TTS (Dougherty *et al.*, 2019). In contrast, suprathreshold ABR wave-1 amplitude reductions were less consistent in chinchillas. The relative diagnostic strengths of MEMR and ABR assays were consistent with parallel studies of noise-exposed and middle-aged humans (Dougherty *et al.*, 2019). These types of studies could have significant translational research value and can pave the way for innovative approaches to evaluate hearing loss using reliable, non-invasive and objective methods.

### D. Predicting PTS based on noise-exposure parameters and individual TTS

The question of whether or not the degree of TTS after noise exposure can predict PTS has been investigated extensively (Mills, 1973; Saunders *et al.*, 1977; Hamernik *et al.*, 2002). TTS is defined as a shift in threshold from baseline levels within some short time frame after the onset of the noise exposure that either 1) resolves entirely within 12–48 h post noise exposure, 2) resolves to some degree and results in a small PTS, or 3) does not resolve (i.e., PTS). Studies as early as the 1950s briefly subjected humans to TTS in tightly controlled settings, but the risk of PTS shifted the bulk of research in this area to the laboratory animal realm. As data began to surface in support of a relationship between noise parameters, TTS, and PTS, researchers looked at the viability of the chinchilla as a research subject. One early study exposed two chinchillas to varying levels of noise (from 70 to 90 dB SPL) at intervals interspersed with behavioral threshold testing (Peters, 1965). This study concluded that (1) the chinchilla is slightly more susceptible to TTS than humans, (2) their TTS recovery pattern mirrors humans and cats, and (3) ultimately, the chinchilla is a good candidate for TTS/PTS investigation. Following this publication, several studies focused their efforts on understanding TTS and PTS using the chinchilla.

Clark and Bohne (1978) used various noise exposures to take a closer look at anatomical correlates of TTS and PTS and modeled the experiment in an attempt to mimic the 4 kHz “noise notch” that is classically present in humans with NIHL. The group that was exposed to nine days of 95 dB SPL narrow-band noise (CF = 500 Hz) generally showed 20–40 dB of TTS with 30% OHC loss, though one animal experienced a PTS of 21 dB centered at ∼4 kHz. The second group, exposed to 18 days of 95 dB SPL narrow-band noise (CF = 500 Hz), had 30–50 dB TTS, no low-frequency PTS, and up to 30 dB PTS in the high frequencies. The second group experienced similar OHC loss (30%). The authors concluded that the broad loss of OHCs may not always translate to corresponding PTS (Clark and Bohne, 1978), which has been corroborated in later studies.

Subsequent studies used surgically monaural chinchillas to investigate TTS during and after nine days of continuous 4-kHz centered narrow-band noise presented at different levels (80, 86, 92, and 98 dB SPL; 57, 65, 72, 80, 86, and 92 dB SPL) (Mills, 1973; Saunders *et al.*, 1977, respectively). Behavioral thresholds were measured beginning 4 min post-exposure onset and then periodically afterwards. These studies documented a decline in TTS up to 90 days after the initial exposure. As the noise exposure intensity level increased, so did the TTS and PTS; unsurprisingly, the group exposed to the highest noise level (98 dB SPL) experienced the highest degree of TTS and therefore the most PTS. The authors constructed equations to predict threshold shift at asymptote during the noise exposure at several frequencies based on the initial noise-exposure level, frequency, and duration of exposure.

Noise-exposure models have been developed over the years, such as the equal-energy theory implemented by the International Standards Organization (ISO, 1975) and the time-weighted average theory proposed in the original NIOSH Criteria document (NIOSH, 1972). Another chinchilla study aggregately evaluated these models by investigating how intermittence in continuous noise exposure (i.e., noise exposures with short breaks of minutes to an hour) affected these models' ability to predict damage and PTS (Ward, 1991). The reduction in damage observed with intermittence was not predicted by the equal-energy model but was over-predicted by the time-weighted average theory. This study is one example where different levels and durations of noise exposure across chinchilla experiments have been combined in an attempt to define the boundary between temporary and permanent hearing loss and to develop more accurate predictive formulae for NIHL.

Finally, after looking at the body of research comprising TTS and PTS, Hamernik *et al.* (2002) incorporated frequency effects into the noise TTS/PTS model and created an equation to predict PTS based on the degree of TTS at a given frequency. They concluded that measuring TTS 24 h after a given noise exposure affords an accurate depiction of the extent of peripheral damage, and noted that any degree of TTS at 24 h post exposure will predictably result in some amount of PTS.

In addition to the animal model itself, the information gained through multiple experiments can be used in order to create computational models of NIHL. Computational models are important in order to reduce the number of animals used for auditory experiments. Recently, Chan *et al.* (2016), developed a new impulse-noise-injury model, Auditory 4.0, to predict the degree of TTS and PTS, as well as TTS recovery, from exposures in unprotected ears. This empirical model was derived based on previous TTS and PTS data from chinchillas exposed to impulse noise, plus new chinchilla data on TTS recovery. Auditory 4.0 is the first model to predict the full range of TTS and PTS dose-response curves based on A-weighted sound exposure level, as well as TTS recovery. Human predictions were made by simply applying a data-driven 28-dBA level shift to account for the higher noise-susceptibility of chinchillas as compared to humans. This model was validated based on previous rifle-noise tests, and is helpful for the armed forces in order to better predict the effects of firearms noise on unprotected ears.

### E. Effects of impulse and impact noise on noise-induced hearing loss

Perhaps equally as important as detailing the effects of long-term noise exposure on the auditory system, it is also crucial to examine the effects of brief traumatic noise exposures in the cochlea. The military has a long history with the effects of impulse and impact noise. As of 2014, over 900 000 veterans receive compensation for service-connected hearing loss, due to repeated exposure to weapons fire and blast explosions (VBA, 2014). Using animal models to understand better the nature of damage done to the auditory system could help develop protective measures for active duty military members and reduce the burden of NIHL on returning veterans. Additionally, impulse and impact noise can create symptoms beyond hearing loss, including tinnitus, the number-one service-connected disability, dizziness, and post-traumatic stress disorder (Yong and Wang, 2015); however, this section will focus on the consequent threshold shifts and histological damage.

Exposure to very short duration, high-intensity acoustic signals is more damaging to the ear than exposure to continuous Gaussian noise (Suvorov *et al.*, 2001). Impact and impulse noises are both very short-duration (<1 s duration), high-intensity stimuli that are characterized by sharp rises in intensity followed by steep declines. Impact noises are generated by two solids coming into contact and are generally less utilized as a noise source in literature, especially due to the ease with which impulse noises are created. Impulse noises are produced by swiftly releasing compressed air from a shock tube; a classic real-world example of impulse noise is gun fire. Many of the high-level repeated impulse-noise exposures used were specifically modeled after M16 rifle fire (e.g., 50 pairs of impulses presented 50 ms apart at 150 dB SPL, Henselman *et al.*, 1994).

Initial impulse-noise-damage studies examined the extent of histological damage in the chinchilla cochlea and came to some interesting conclusions. First, in one study (Henderson *et al.*, 1974), 155 dB peak SPL impulse exposed animals displayed uniform and complete loss of OHCs 8–12 mm from the apex with small losses of IHCs in the same region. However, there was no evidence of PTS 1000 h post-exposure. In contrast, impulse noise exposures of 161 dB SPL produced a wide range of effects with some animals showing robust TTS but no PTS (six chinchillas), whereas others (four chinchillas) showed PTS that ranged from 10 to 30 dB. Animals with PTS showed nearly complete loss of both OHCs (8–18 mm from apex) and IHCs (12–16 mm from apex). A third group was exposed to 166 dB SPL impulse noise. Remarkably, this group showed smaller PTS (10–20 dB) than the 161 dB SPL group. Although there was substantial OHC loss in the basal end of the cochlea, IHC loss was small and scattered. Moreover, the small loss of IHCs was below the level observed in the 155 dB SPL exposed group.

The non-monotonic degree of hearing loss and damage with increasing SPL observed in these data has been attributed to a critical intensity at which there is conductive failure. Early studies in guinea pigs (Akiyoshi *et al.*, 1966) suggested that intensities between 160 and 180 dB SPL can mechanically disrupt middle-ear function, which ultimately provides a degree of damage protection to the inner ear with the tradeoff of transient or permanent middle-ear damage. Middle-ear damage (i.e., rupturing of the tympanic membrane or ossicular chain disturbance), likely contributed to the differences seen in their results; however, the integrity of the middle ear was not assessed in that study. In the 166 dB SPL exposed chinchilla group (Henderson *et al.*, 1974), all animals showed tympanic-membrane rupture, thus reducing the acoustic energy transfer to the cochlea. Tympanic-membrane rupture and other mechanical middle-ear disruption following impact noise were not considered in early damage risk models involving the chinchilla (Rosowski, 1991), which focused more on the effects of external and middle-ear filtering on hypersensitivity at mid-frequencies. It is now clear that tympanic-membrane rupture is crucial to understanding fully and predicting noise risk for extremely high sound levels. Neither intensity level nor total acoustic energy fully account for noise hazard risk, a finding that was further realized in a later model using chinchillas proposed by Hamernik and Qiu (2001).

To account for multiple acoustic variables and middle-ear involvement in auditory hazard risk assessment, a series of studies was compiled in order to create a more accurate model of noise risk to the human ear. The Auditory Hazard Assessment Algorithm for Humans (AHAAH) was proposed by the US Army Aeromedical Research Laboratory using data from human volunteers in a variety of conditions and previous models (Price, 1974; Price and Kalb, 1991; Patterson and Ahroon, 2004; Greene *et al.*, 2017). The AHAAH model included factors such as the transfer function of the external and middle ear, the “warned” vs “unwarned” status of the middle-ear reflex muscle, and stapes displacement to predict auditory hazard risk. For the most part, the model over-predicted risk relative to actual data, but its continual development may provide an extremely useful tool in understanding NIHL. It may be possible to use data garnered from animal models such as the chinchilla to contribute to this model, particularly at extremely high sound pressure levels. For example, recent detailed work on tympanic-membrane motion and damage following blast exposures in chinchillas is likely to be particularly valuable (e.g., Gan *et al.*, 2016; Liang *et al.*, 2017).

Ensuing studies sought to characterize the degree of TTS and PTS that occurs from impulse noise by looking at asymptotic threshold shift (ATS) during long-lasting (e.g., many days) noise exposures (Blakeslee *et al.*, 1978). ATS is a term that isolates the stabilized threshold level that occurs after the initial TTS, but during the noise exposure. The study found that impulse noise provided a higher, more variable ATS faster than continuous-noise exposure, but no conclusions could be made regarding their differences in TTS and PTS. Thus, it appears that long-term continuous exposures provide a better method for examining noise-induced PTS than impulse-noise exposures.

In a more recent study, Hu *et al.* (2006) investigated OHC death following impulse noise exposure (155 dB SPL) in 37 chinchilla cochleas, harvested at different time intervals after exposure. In the study's findings, the noise exposure initiated apoptosis in the OHCs immediately. At five minutes post-exposure (when the organ of Corti was fixed), apoptosis had reached an advanced stage much faster than expected relative to non-impulse noise, perhaps due to direct impulse-induced mechanical trauma.

### F. Effects of intermittent noise exposure on hearing loss

High-level noise exposures produce both mechanical and metabolic damage to sensory and supporting cells in the inner ear. The damage profile can be immediate or delayed, and thus it is not surprising that intermittent noise exposures have been shown to produce different and interesting results, compared with continuous-noise exposures (Eldredge *et al.*, 1959; Kryter *et al.*, 1966; Sataloff *et al.*, 1969; Ward, 1970; Schmidek and Carpenter, 1974; Clark *et al.*, 1987). The value of studying intermittent noise exposures is that noise exposure in humans tends to be intermittent, whether it is recreational or occupational. Studies in humans and animals have shown that intermittent noise exposures produce less hearing loss than continuous exposures, even when level and total exposure time are equivalent. These findings seemingly violate the equal-energy hypothesis that threshold shift and cochlear damage are the result of the level and duration of a noise exposure (e.g., Harding and Bohne, 2004).

The underlying mechanisms for why intermittent-noise exposures produce less PTS and cochlear damage have been of intense interest, as these could elucidate important factors in susceptibility to noise exposure. In chinchillas, a number of studies have looked at the effects of intermittent noise exposure (e.g., Clark *et al.*, 1987; Ahroon and Hamernik, 1999). In experiments looking at intermittent 95 dB SPL noise exposures lasting 6 h/day for 36 days or 15 min/h for 144 days, thresholds were found to increase initially by 35–45 dB, but returned to within 10–15 dB of baseline within a few days of starting the long-term exposure (Clark *et al.*, 1987). Behavioral data on hearing sensitivity as well as anatomical results showed less PTS and less cochlear damage than continuous noise exposures with the same energy. Similarly, 95 dB SPL noise exposures for 15 min/h for 4 or 40 days produced the same pattern of results as well as recovery of single-fiber tuning curves by day 40 (Sinex *et al.*, 1987). These findings in the chinchilla suggest that adaptation and resilience to noise exposures occur, and that this effect originates in the peripheral auditory system.

### G. Ear toughening, effects of previous noise exposures

**FIG. 4.**
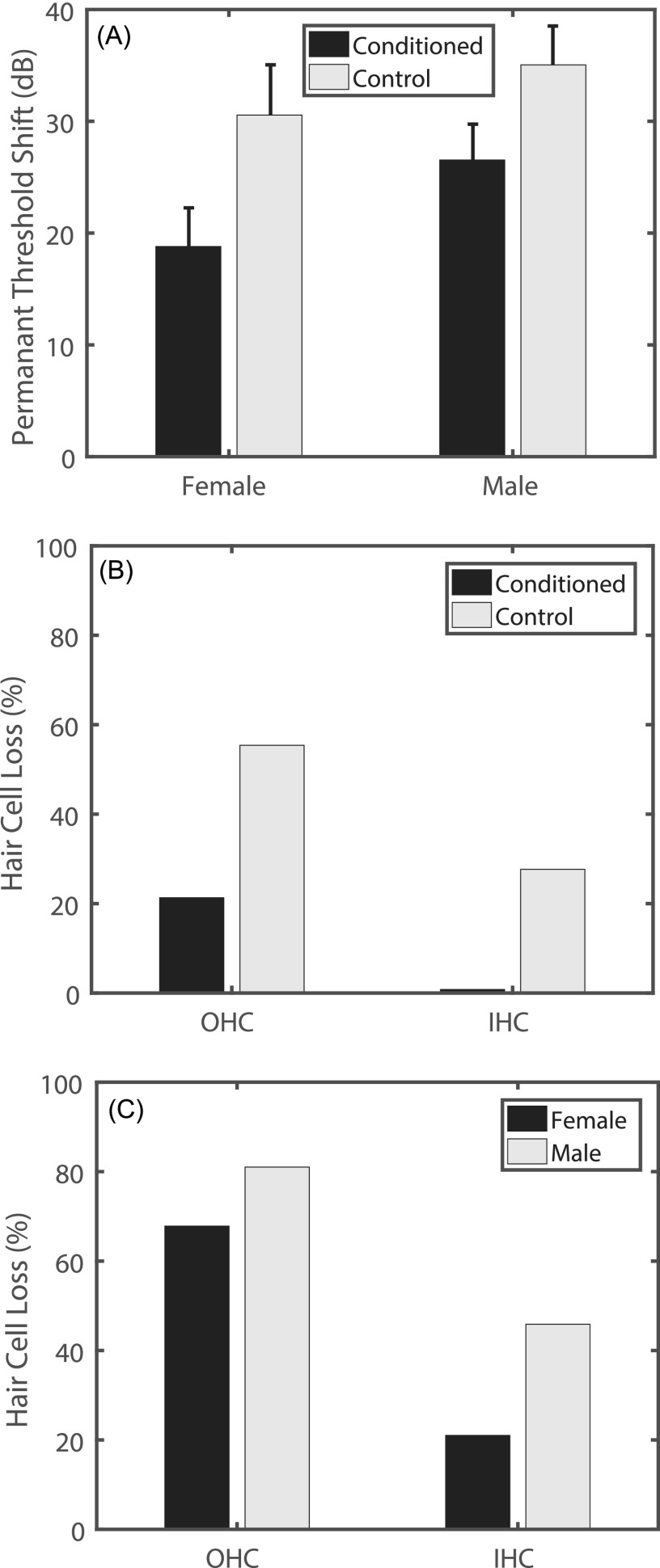
Chinchilla data demonstrate that PTS and hair-cell loss due to high-level simulated rifle fire (impulse noise) is reduced when preceded for several days by moderate-level conditioning noises, and that there are sex differences in susceptibility to noise exposure. (A) Permanent threshold shifts at low frequencies (0.5, 1, and 2-kHz) were less in both sexes when preceded by 95 dB SPL, 0.5-kHz octave-band noise played for 6 h/day for five days. Data replotted with permission from McFadden *et al.* (2000). Copyright 2000, Acoustical Society of America. (B) Hair-cell loss after simulated rifle fire was reduced in the conditioned case for both OHCs and IHCs. Cochleogram data (Henselman *et al.*, 1994) were averaged from 0.5 to 2 kHz, and plotted with permission from Hearing Research. (C) Hair-cell loss after simulated rifle fire was less for females than for males. Cochleogram data (McFadden *et al.*, 1999) were averaged from 0.5 to 2 kHz, and plotted with permission from Ear and Hearing.

The phenomenon of resistance to the damaging effects of noise exposure resulting from intermittency or previous prolonged lower-level exposures has been termed “conditioning effects,” “priming effects,” or “ear toughening” [Figs. 4(a) and 4(b)], and has been studied extensively in chinchillas (Subramaniam *et al.*, 1992; Henselman *et al.*, 1994; Ahroon and Hamernik, 1999; McFadden *et al.*, 2000). Chinchilla studies have been particularly important in characterizing the dependence of protective conditioning effects on the parameters of prior noise exposure.

One question of interest was whether the number of days of prior exposure to a lower-level conditioning noise affected the protective effects for subsequent higher-level exposures. To evaluate this question, PTS following a 48-h exposure to 106 dB SPL noise was compared between non-conditioned chinchillas and those exposed to a lower-level conditioning noise (95-dB SPL, 0.5-kHz octave-band noise played for 6 h/day) for 1, 10, or 20 days. Each of the conditioned groups showed significantly less PTS than the non-conditioned groups, with no significant differences in PTS (or hair-cell damage) between the 1-, 10-, and 20-day groups (Subramaniam *et al.*, 1993).

Other variables related to the conditioning effect have also been studied in chinchillas, such as the duration of the conditioning effect (McFadden *et al.*, 1997b). Chinchillas were exposed to low-frequency conditioning exposures (0.5 kHz octave-band noise, 90–95 dB SPL, 6 h/day for 10 days) and subsequently exposed to a higher-intensity exposure (106 dB SPL, 0.5 kHz octave-band noise for 48 h). Relative to controls, exposed only to the higher-level noise, the conditioned animals had significantly lower PTS. This protective effect lasted longer than two months. Thus, it appears that conditioning can produce long-term protective effects as opposed to short-term physiological protection.

Conditioning effects have also been shown to extend to different types of noise exposures, such as rifle fire. Chinchillas conditioned with noise (95 dB SPL, 0.5 kHz octave-band noise, 6 h/day for 5 days) showed less PTS and hair-cell loss [Fig. 4(B)] after a subsequent impulse-noise exposure (50 pairs of 150 dB SPL impulse noises, 50 ms apart, 1 s between pairs) relative to controls only exposed to the impulse noise (Henselman *et al.*, 1994).

In summary, chinchilla conditioning experiments have demonstrated the following: (1) the ear can indeed be toughened by lower-level pre-exposures; (2) these effects can be long-lasting; and (3) these effects convey protection against other types of noise such as impulses. It is important to note that the precise mechanisms underlying these “conditioning” effects are not well understood. However, conditioning studies have provided initial insight into underlying mechanisms that has led to investigations into potential strategies for prevention and rescue.

In chinchillas and other species, noise exposures result in increased levels of reactive oxygen species (ROS) as a metabolic byproduct of high levels of cochlear activity during noise. The high levels of ROS have been shown to potentiate damage to the inner ear; however, ROS activity can be mediated by endogenous antioxidant activity. When antioxidant enzyme levels were compared between conditioned animals (with less hearing loss) and non-conditioned animals (with more hearing loss), conditioning was shown to enhance the endogenous antioxidant glutathione (GSH), in addition to catalase (Jacono *et al.*, 1998). These results have been interpreted to suggest that GSH system-enhancing agents could act to protect the ear against noise exposure (as discussed further in Sec. IV).

Furthermore, ear-toughening experiments are particularly interesting given the current state of science as it pertains to synaptopathic hearing loss (Hickox *et al.*, 2017). A closer review of ear-toughening experiments shows that in some cases thresholds experience minimal change (Henderson *et al.*, 1984), but it is now clear that significant cochlear damage can occur in the absence of PTS (Kujawa and Liberman, 2009). In other experiments, thresholds are increased, but there are no observed differences in cochlear damage (e.g., Clark, 1991). Current and future studies looking at thresholds, hair-cell loss, and synaptic loss will likely provide a more complete characterization of these conditioning effects and provide important insights towards prevention and rescue strategies. The chinchilla provides an excellent model for these types of studies, where comparisons of anatomical, physiological, and behavioral effects will be essential.

### H. Effects of industrial solvents on noise-induced hearing loss

In the section above, the effects of previous noise exposure were shown to reduce subsequent NIHL. However, NIHL can also be augmented. Industrial solvents such as toluene and styrene have been repeatedly shown to potentiate NIHL in animal models (Hodgkinson and Prasher, 2006; Chen *et al.*, 2008; Lund and Kristiansen, 2008; Chen and Henderson, 2009; Rumeau *et al.*, 2011). In chinchillas, exposure to toluene, a widely-used industrial solvent, yielded no significant ototoxic effects by itself or when combined with noise exposure, unlike in rats and humans (Davis *et al.*, 2002). The study further hypothesized that chinchilla metabolism and P450 enzymatic activity in the liver mediate the ototoxic effects of toluene to a greater extent than in rats, mice, and humans (Davis *et al.*, 2002). The differences seen between chinchillas and humans indicates that the chinchilla may not be the ideal model to investigate the effects of solvents in combination with noise exposure; however, they may be useful in studies to understand biological mechanisms for how ototoxic agents can be broken down into less toxic compounds.

### I. Effects of aging and noise exposure

It is well established that 1 in 3 adults in the United States over the age of 65 develop some degree of hearing loss (Lin *et al.*, 2011). The complex aging auditory system is plagued by an array of deficits including: the mechanical structures and metabolic processes in the cochlea, the stability of the AN pathway, and the integrity of the central auditory nervous system. The mechanisms that culminate in age-related hearing loss are a composite interaction between genetics, environment, sensory integration, and neural networks; researchers have looked closely at each of these effects in order to better understand the resulting hearing difficulties in humans. The relationship between presbycusis, or age-related hearing loss and noise exposure has been frequently acknowledged in the literature. There is a positive trend between individuals who are more regularly exposed to damaging noise levels, whether occupationally or recreationally, experiencing a greater degree of hearing loss as they age (Gates *et al.*, 2000). However, analyzing the multifaceted effects of age on hearing ability requires more investigation.

Numerous studies have created animal models to examine age-related hearing loss, most commonly using the Mongolian gerbil (Schmiedt *et al.*, 2002) and the C57 strain of mouse (Johnson *et al.*, 1997). Some of these animal models (e.g., the C57 mouse) are genetically bred to undergo swift, progressive, and profound SNHL, making experimental studies more efficient. The chinchilla lacks this genetically deliberate age-related hearing loss; however, as discussed previously, the chinchilla's genetic diversity makes them a better-suited model to humans, and thus for pre-clinical studies. Additionally, the chinchilla offers a unique adaptation of the age-related hearing loss animal model due to their comparative longevity (∼8–20 years) in relation to the Mongolian gerbil (∼5 years) and C57 mouse (∼2 years). This longer lifespan allows researchers to track hearing over the course of a chinchilla's lifetime and ideally draw more accurate conclusions.

A handful of studies have looked at the aging chinchilla auditory system. Bohne *et al.* (1990) found through cochlear histology that sensory, neural, and strial cochlear pathology begins at three years of age and progress as chinchillas get older. A second study observed that the pattern of age-related hearing loss exhibited by chinchillas closely matches that of humans, such that the high frequencies are affected first and experience the most sensory loss (McFadden *et al.*, 1997a). This same study also determined that the chinchilla loses hearing sensitivity at a rate of 0.8–2 dB hearing level (HL)/year, a rate that closely matches humans (0.2–2 dB HL/year). Thus, the aging chinchilla auditory system draws parallels to humans, and serves as a unique model to examine it more closely and in more specific contexts. Introducing noise to this model has had interesting outcomes. Sun *et al.* (1994) exposed aging (from 8 to 13 years old) and young (from 8 to 12 months old) control chinchillas to a 95 dB SPL continuous narrow band noise (CF = 0.5 kHz), and found no significant differences between the two age groups when looking at OHC and IHC loss. Though the study did not examine functional hearing ability between the two chinchilla groups, these results suggest that though noise exposure may accelerate age-related hearing loss within a lifetime, subsequent noise exposure to an aged auditory system does not cause additional tissue damage in the chinchilla. The relationship between hazardous noise and age-related hearing loss is still under investigation. As it stands, the chinchilla serves as an excellent model to continue research in this area.

### J. Sex differences in susceptibility to noise induced hearing loss

Isolating sex differences in humans is challenging for reasons that are not exclusive to noise research. Environmental differences that develop over individual lifetimes are impossible to control in all areas of human study. More specifically, it is difficult to isolate sex differences in NIHL research because historically, occupations that involve noise exposure are male-dominated (e.g., military, manufacturing, construction), though female representation has slowly increased over time. As an example, as of April 2018, women represent just 16% of the US Military, a number that shows significant increase from the 2% reported in 1973 (Reynolds, 2018). The overall increase of women in the workforce invites more investigation on whether or not sex plays a role in the extent of NIHL within an individual.

Recent large-scale prospective cohort studies have looked at the differences in average hearing loss of adults across age and sex and compared the results to previous studies that had done the same (Hoffman *et al.*, 2017; Homans *et al.*, 2017). These studies found that men had more hearing loss than women, but the results were significantly less pronounced than the results of previous studies; according to the researchers, societal changes and equalizing experiences were likely to blame for the shift. Nonetheless, the long-established bias between male and female NIHL has raised the question of whether or not males may be more susceptible than females to NIHL (Ward, 1966; Engdahl and Tambs, 2010). Additionally, as of 2015 the NIH adjusted its policy to require the consideration of sex as a biological variable for all NIH-funded pre-clinical research studies, inviting further investigation regarding differences in vulnerability to NIHL between sexes.

Overall, chinchillas have been used sparingly as an animal model in this context. One such study exposed a group of male and female chinchillas to an impulse noise reaching approximately 150 dB SPL, then re-assessed their auditory sensitivity at various intervals post exposure to determine TTS and PTS levels (McFadden *et al.*, 1999). Cochlear histology was completed to confirm anatomical damage. Prior to exposure, this study found that there were minimal sex differences in threshold and waveform amplitude determined by IC evoked potentials. The female chinchillas were found to have slightly higher low-frequency thresholds, and slightly lower high-frequency thresholds than the males. DPOAE amplitudes showed no significant differences between males and females. Immediately post exposure, both genders exhibited significant TTS; however, the female chinchillas experienced less permanent low-frequency hearing loss and hair-cell loss than the males [Fig. 4(C)], but more high-frequency hearing loss (McFadden *et al.*, 1999). Though the results are of interest, they lacked conclusiveness in part due to the magnitude of the effects and because the females had lower pre-exposure high-frequency thresholds than the males.

A follow up study explored differences between male and female chinchillas in the effects of a 95 dB SPL sound conditioning paradigm prior to exposure to 150 dB SPL impulse noise designed to simulate M16 rifle fire (McFadden *et al.*, 2000). The authors found that the females experienced significantly more TTS following sound conditioning. However, relative to the males, these same females showed less PTS in the low-frequency region where pre-exposure thresholds were comparable between sexes [Fig. 4(A)]. Consistent with the previous study [Fig. 4(C)], these authors determined that female chinchillas also exhibited less IHC and OHC loss than male chinchillas.

Collectively, these studies suggest that there may be small but significant (potentially frequency-specific) differences that could make males more susceptible to NIHL than females. These differences have been seen in anatomical damage as well as functional hearing ability, but an understanding of why these differences might exist is not yet clear. The results found in the studies mentioned parallel the nominal human research addressing the same topic and highlights the chinchilla as an acceptable animal model to further investigate sex differences in NIHL.

### K. Suprathreshold behavioral effects of noise exposure

Evidence continues to accumulate that threshold-shift effects of hearing loss only partially account for the perceptual effects that people with hearing loss face in daily life, particularly in noisy environments. Thus, as NIHL and prevention/rescue studies advance, it will become all the more important to consider suprathreshold effects of noise exposure on behavior and neural coding of perceptually relevant sounds, in addition to anatomical and physiological effects. Inasmuch as behavioral techniques have been largely replaced with objective physiological measures for threshold effects, for experiments that use complex auditory stimuli (e.g., listening in noise or other tasks), operant behavioral techniques may be more appropriate where objective physiological measures may not adequately assess study outcomes.

Chinchillas have long been used successfully to evaluate normal-hearing behavioral acuity using complex stimuli (e.g., Salvi *et al.*, 1982; Saunders *et al.*, 1987; Shofner, 2000; Shofner *et al.*, 2019). For this reason, chinchillas have the potential to evaluate numerous perceptual effects of noise exposure. As briefly discussed previously, and in detail by Radziwon *et al.* (2019) in this special issue, the effects of noise exposure on temporal acuity as measured by gap-detection and amplitude-modulation-detection tasks have been shown to be reduced in a similar way to humans (Giraudi-Perry *et al.*, 1982; Henderson *et al.*, 1984). In more recent studies on the perceptual effects of noise-induced cochlear synaptopathy, chinchillas did not show a substantial overall deficit in modulation detection with tonal carriers following moderate TTS-inducing noise exposure (Maulden *et al.*, 2017). These results suggest that there is a critical boundary for the emergence of perceptual deficits. In contrast, chinchillas with carboplatin induced selective IHC loss (but minimal threshold shifts), were shown to have degraded tone detection in noise (Fig. 3; Lobarinas *et al.*, 2016). The results of these chinchilla studies highlight the complex relationship among anatomy, physiology, and auditory perception. For these reasons, chinchillas are an excellent model to explore the effects of hearing loss on tasks requiring more complex stimuli, particularly in cases where there is no PTS.

### L. Effects of noise exposure on neural coding

A number of studies have used chinchillas to explore the effects of noise exposure on fundamental aspects of neural coding, motivated by psychophysical studies focused on human listeners with hearing loss. Numerous psychophysical studies have found that listeners with cochlear hearing loss may be unable to track the rapid fine-structure cues that are necessary for speech or pitch perception (reviewed by Moore, 2008). A series of noise-exposed chinchilla studies has explored the physiological basis for this psychophysical phenomenon (reviewed by Henry and Heinz, 2013).

One hypothesis inferred that the psychophysical effect arises from degraded AN-fiber phase locking; however, this was disproven as AN-fiber phase locking in quiet conditions remained unchanged following noise exposure (Kale and Heinz, 2010). This result suggests that the fundamental ability of AN fibers to encode rapidly varying fine-structure cues is not degraded in AN fibers. In contrast to quiet conditions, AN-fiber phase locking to tonal stimuli was degraded in background noise; an effect that was associated with broader tuning (i.e., allowing more of the background noise to degrade temporal coding; Henry and Heinz, 2012). This result was important because it confirmed that degradations in temporal fine-structure coding do exist in the auditory periphery, but primarily in noisy situations where listeners with SNHL have the most difficulty.

Neural coding of slowly-varying envelope modulations in chinchilla AN fibers was shown to be enhanced following noise exposure, likely due to the combined effects of OHC and IHC dysfunction (Kale and Heinz, 2010). Envelope coding in the chinchilla central auditory nervous system also appeared to improve following noise exposure based on frequency-following-response evoked potentials measured at the scalp (Zhong *et al.*, 2014). Enhanced neural envelope coding following noise exposure is consistent with improved modulation detection in some human hearing-impaired listeners (Moore *et al.*, 1996). It is possible that this enhancement produces more distracting fluctuations due to background noise for hearing-impaired listeners compared to normal-hearing listeners, and thus may contribute to difficulties listening in everyday situations with cochlear hearing loss.

Finally, a dramatic effect of noise exposure on the tonotopic representation of complex sounds was observed in chinchilla AN fibers based on advanced system-identification analyses of broadband-noise responses (Henry *et al.*, 2016). Following only moderate hearing loss, mid-frequency AN fibers (CFs ∼ 2–4 kHz) that typically encode the envelope of complex sounds, began to encode the temporal fine structure of low-frequency (non-tonotopic) components of the broadband noise. In general, this description of distorted tonotopic representation of complex sounds is consistent with degraded vowel coding after noise exposure, a phenomenon that has been thoroughly characterized in cats (Miller *et al.*, 1997). In general, distorted tonotopy has significant implications for most neural coding theories of auditory perception of complex sounds. Interestingly, distorted tonotopy is much more prevalent for a NIHL than for a metabolic (e.g., age-related) hearing loss of similar degree (Henry *et al.*, 2019), further highlighting the importance of avoiding noise overexposure.

### M. Noise-induced central plasticity

Thus far, this review has focused primarily on the effects of NIHL on the peripheral auditory system, but many studies have carefully studied central auditory nervous system responses to noise exposure. As mentioned previously, patients with SNHL typically have difficulty perceiving speech, particularly in the presence of background noise. Often patients with SNHL also experience loudness recruitment, a phenomenon where loudness grows more rapidly than normal as sounds get more intense. These symptoms are not always immediately measurable in current audiological diagnostic testing, as patients with similar pure-tone thresholds may exhibit vastly different suprathreshold functional capabilities. Based on current chinchilla studies, noise-exposure damage affects more than just the peripheral auditory system and extends into the central auditory pathway where plasticity is affected (e.g., Kim *et al.*, 1997; Morest *et al.*, 1997; Morest *et al.*, 1998). The degree of damage and the corresponding functional deficits are still under investigation as researchers seek to isolate sites of lesion in the central auditory nervous system.

Early research done by Salvi and Ahroon (1983) used an 86 dB SPL continuous-noise exposure on chinchillas in order to evaluate possible neural correlates of tinnitus in high-frequency hearing loss. The study evaluated two-tone inhibition (TTI) and spontaneous activity for individual AN fibers. The significant findings of this study were that the noise-exposed AN fibers had frequency-specific increased spontaneous rates, and diminished TTI and hearing sensitivity. The authors suggested that the increased spontaneous rate could be more evidence in support of the “edge” theory of high-frequency, high-spontaneous rate fibers contributing to tinnitus, and implying that noise exposure alters remaining nerve fiber function to some degree.

Following the tinnitus model and delving further into the central auditory nervous system, successive studies scrutinized the IC in the auditory midbrain. The IC is thought to play an important role in sound localization processing within the auditory pathway (Langner *et al.*, 2002; Jones *et al.*, 2013). Salvi and colleagues (Salvi *et al.*, 1990) exposed chinchillas to a 105 dB SPL 2-kHz pure tone and measured the amplitude of the IC evoked response. Predictably, the results showed significant PTS in the IC response, a reduction of overall response amplitude, and OHC loss (10%–50%, depending on the region of the cochlea). Interestingly, as the intensity of the stimuli was increased during IC evoked response measurements, IC amplitude rapidly surpassed pre-exposure amplitudes when testing in the region of hearing loss. These enhanced evoked potentials could underlie loudness recruitment. Another study further investigated this phenomenon and summarized that IC neurons experience hyperactivity post lesion (Wang *et al.*, 2002). This hyperactivity culminates in broader frequency responses, higher spontaneous discharge rates, and enhanced response amplitudes. The authors postulated that after a lesion, lateral-inhibitory circuits are lost and fail to modulate excitatory responses. Other findings in this study include the effects of selective IHC loss on evoked responses. This study found that the compound action potential (CAP) was reduced in amplitude, while IC response amplitude and frequency selectivity remained unchanged, consistent with the notion of a compensatory central gain mechanism playing a role following NIHL (Salvi *et al.*, 2000). Given their extensive history of noise-exposure studies, and their advantages in collecting robust data across the entire auditory system, chinchillas provide an excellent animal model to further explore central effects of NIHL.

## IV. CHINCHILLAS AS A PRE-CLINICAL MODEL FOR PHARMACOLOGICAL RESCUE AND PREVENTION STUDIES

NIHL can result from excessive noise exposure occurring in occupational or recreational settings. This exposure can be continuous and expected (e.g., working on the flight deck of an aircraft carrier) or can be impulsive and unexpected (e.g., from improvised explosive devices). Occupational hearing conservation programs exist to monitor hearing and to provide hearing protection devices (HPDs) to prevent long-term hearing loss. However, in situations such as military operations, these HPDs can be either impractical or ineffective for the types of acute acoustic trauma (AAT) experienced in the field. Current military protocols or treatments for AAT are largely impractical (e.g., quiet environment for many days) and/or are not completely effective or are associated with serious side effects (e.g., steroids, vasodilators, and hyperbaric oxygen therapy aimed to improve inner-ear oxygen and blood flow). Thus, pharmacological strategies are of great interest for protection and rescue from NIHL. A number of pharmacological agents have been motivated by ear-conditioning experiments (many in chinchillas, see Sec. III G), which suggest that enhancing endogenous antioxidant activity can make the inner ear more resistant to noise exposure (Jacono *et al.*, 1998).

Pharmacological enhancement of the endogenous antioxidant GSH system by administration of systemic antioxidants has been shown to attenuate the damaging effects of noise in rats, mice, and chinchillas (Campbell *et al.*, 2007). In chinchillas, the administration of D-methionine (D-met) two to three days prior to noise exposure reduced both noise-induced (105 dB SPL, 4-kHz centered, octave-band noise, 6 h) PTS as well as OHC loss (Claussen *et al.*, 2013). These results suggest that pre-loading D-met with no post-exposure administration may provide a measure of protection to the inner ear. The potential for D-met to be used in rescue scenarios has also been explored in chinchillas (Campbell *et al.*, 2011). The effectiveness of D-met administered 3 to 7 h post noise exposure was evaluated in terms of ABR threshold shifts and OHC survival. Chinchillas were exposed to octave-band noise presented at 105 dB SPL for 6 h, and then received intraperitoneal injections of D-met or saline (control) after the exposure. ABRs were measured pre-exposure, 1-day post, and 21-days post exposure. The results of this study showed that when D-met was administered 3, 5, or 7 h post noise exposure, ABR thresholds significantly reduced at 2 and 4 kHz, and OHC loss was also reduced at the 2, 4, 6, and 8 kHz regions of the cochlea. These findings demonstrate that some rescue from noise-induced PTS is possible even if D-met administration is delayed for up to 7 h after noise exposure.

**FIG. 5.**
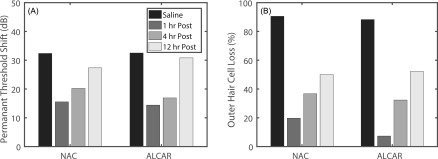
A time-dependent therapeutic window exists in chinchillas for protecting against noise-induced PTS (A) and outer-hair-cell loss (B) for the administration of the antioxidant compound N-acetylcysteine (NAC) and for the mitochondrial protectant acetyl-L-carnitine (ALCAR). Both PTS and hair-cell data were averaged from 2 to 8 kHz, and replotted with permission from Coleman *et al.* (2007a).

Other compounds to treat AAT and auditory hair-cell damage after noise exposure have also been explored in chinchillas (Coleman *et al.*, 2007a). One such compound was N-acetylcysteine (NAC), which has the ability to counteract cochlear oxidative stress caused by the loss of cellular antioxidants and the production of ROS. In previous animal studies, the use of NAC has been shown to replenish the major cellular antioxidants and GSH in hair cells (Ohinata *et al.*, 2000). A second compound used was acetyl-L-carnitine (ALCAR), which acts as a cellular protectant by maintaining mitochondrial integrity. Chinchillas were exposed to 6 h of 105 dB SPL octave-band noise centered around 4 kHz (Kopke *et al.*, 2000; Coleman *et al.*, 2007a). Animals were initially injected intraperitoneally 1, 4, or 12 h post noise exposure with either 325 mg/mL of NAC or 100 mg/mL of ALCAR; control animals were injected with saline. After the initial injection, animals were given injections twice daily for two days. Evoked potentials were measured pre-exposure, immediately after exposure, 11 days, and 21 days post exposure. Results showed that both compounds significantly reduced PTS [Fig. 5(A)] and hair-cell loss [Fig. 5(B)] when administered 1 or 4 h post noise exposure, but did not provide protection from threshold shift when given 12 h post exposure at the doses given. This study showed that a time-dependent therapeutic window does exist for protecting against PTS and hair-cell loss by using NAC or ALCAR in isolation, and suggests that potentially greater and/or longer effects could be obtained from higher doses and/or combined administration of these two compounds (Coleman *et al.*, 2007a).

The role of antioxidant protection has been evaluated in several additional studies with chinchillas. Noise-induced (105 dB SPL, 4 kHz octave-band noise, 6 h) neural degeneration at the level of the cochlear nucleus has been reduced as a result of administering a combination of the antioxidants NAC and ALCAR (Du *et al.*, 2012). Similarly, orally administered antioxidants [4-hydroxy alpha-phenyl-tert-butylnitrone (4-HOPBN) and NAC], given 4 h post noise exposure (105 dB SPL, octave-band noise centered at 4 kHz, 6 h), significantly reduced PTS and OHC damage in a dose-dependent manner (Choi *et al.*, 2014). Whereas some studies have found similar protective effects of antioxidants (Kopke *et al.*, 2000; Kopke *et al.*, 2005; Bielefeld *et al.*, 2007; Coleman *et al.*, 2007a), other studies have shown that following high-level (105 dB SPL), long duration (8 h/day for five days), broadband noise, there is little to no protection by NAC (Hamernik *et al.*, 2008).

Other pharmacological agents assessed in noise-exposed chinchillas have also demonstrated protective effects. AM-111, an otoprotective peptide that inhibits Jun N-terminal kinase (JNK) mediated apoptosis, reduced PTS to 155 dB SPL impulse noise from 16–25 dB to 6–17 dB after a single administration at 1 to 4 h post-exposure (Coleman *et al.*, 2007b). Other apoptotic pathways such as the Src protein tyrosine kinase (Src-PTK) signaling cascade have been implicated in NIHL and have been studied in chinchillas. In two experiments, three Src-PTK inhibitors were evaluated (Harris *et al.*, 2005) on noise-exposed chinchillas (4 h, 4 kHz octave-band noise at 106 dB SPL). All three inhibitors (KX1-004, KX1-005, and KX1-174) showed some degree of protection. In particular, KX1-004 was found to be the most effective in both exposures to the continuous noise and a follow up study using impulse noise (Bielefeld *et al.*, 2011). Testing with additional Src-PTK inhibitors showed that KX2-329 demonstrated significant protection from impulse noise.

These and other studies using chinchillas show evidence supporting the potential for pharmacological rescue after noise exposure, and suggest that a simple oral pharmacological agent could be given several hours post noise exposure and still have widespread clinical utility. Clinical trials are required to explore this possibility in humans given species differences in metabolism (Davis *et al.*, 2002), but chinchillas will remain a useful pre-clinical model for these types of studies evaluating the potential of existing and future compounds to protect the ear following noise exposure.

## V. SUMMARY

The chinchilla has an impressive and long (>50 year) history as a valuable animal model for hearing science and NIHL. They are especially useful when comparisons of anatomical, physiological, and behavioral effects in the same species are important. Chinchillas are relatively easy to train, their docile nature permits awake physiological measures, their anatomy allows for direct physiological measurements along the entire auditory pathway, and their lifespan allows for long-term studies. Thus, the chinchilla model is flexible in accommodating both short- or long-term noise exposures as well as for studies employing continuous, impulse, or impact noise. Furthermore, potential pharmacological treatments can be delivered locally through a relatively accessible and thin round-window membrane, via intraperitoneal or intravenous injection, or orally. As reviewed here, these advantages of the chinchilla model have been and will continue to be effectively leveraged for research on the basic science of NIHL, the perceptual consequences of NIHL, and for evaluating potential therapeutic treatments to reduce or abolish NIHL.

## Footnotes

^1^ https://www.usaarl.army.mil/pages/publications/techreports/ (Last viewed 8/12/2019)

^2^ https://discover.dtic.mil/ (Last viewed 8/13/2019)
